# Distinctive microbiota distribution from healthy oral to post-treatment apical periodontitis

**DOI:** 10.3389/fcimb.2022.980157

**Published:** 2022-09-08

**Authors:** Jing-Lin Zhang, Juanli Yun, Lin Yue, Wenbin Du, Yu-Hong Liang

**Affiliations:** ^1^ Department of Cariology and Endodontology, Peking University School and Hospital of Stomatology & National Clinical Research Center for Oral Diseases & National Engineering Research Center of Oral Biomaterials and Digital Medical Devices, Beijing, China; ^2^ State Key Laboratory of Microbial Resources, Institute of Microbiology, Chinese Academy of Sciences, Beijing, China; ^3^ Savaid Medical School and College of Life Sciences, University of the Chinese Academy of Sciences, Beijing, China; ^4^ Department of Stomatology, Peking University International Hospital, Beijing, China

**Keywords:** post-treatment apical periodontitis, microbiota, next-generation sequencing, metabolic pathway prediction, bioinformation, bacteria

## Abstract

Post-treatment apical periodontitis (PoAP) occurs when root canal treatment has not adequately eliminated bacterial invasion and infection. Yet little is known about the bacterial composition and changes related to the etiology and pathogenesis of PoAP. In this study, clinical samples classified as root apex (HARD) and periapical granulation tissues (SOFT) were separately collected from 10 patients with PoAP. The microbiota of each sample was characterized by 16S rRNA gene sequencing, and the obtained dataset was coanalyzed with 20 NCBI sequence read archive (SRA) datasets of healthy oral (HO) and primary apical periodontitis (PAP). We observed 2522 operational taxonomic units (OTUs) belonging to 29 phyla, and all samples shared 86.5% of the sequence reads. The OTUs affiliated with Bacteroidetes, Firmicutes, Proteobacteria, Fusobacteria, and Actinobacteria, were identified as core microbiota, which accounted for nearly 90% of 16S rRNA sequences in all samples. However, the principal coordinates analysis (PCoA) of the beta diversity demonstrated that the three periapical statuses have distinct microbial compositions. Compared with HO and PoAP, Actinomyces has a significantly increased abundance in PAP. The microbial diversities in PoAP were significantly lower than those in the HO and PAP (p<0.05). The relative abundance of most bacterial taxa was decreasing, except that Clostridia and Synergistia were increased. Furthermore, we explored the potential metabolic differences of the microbial communities by KEGG pathway prediction. We revealed that the microbiota of PoAP might have a more active metabolic capacity, including carbohydrate metabolism, energy metabolism, and enzyme cofactor/carrier biosynthesis (p<0.05). Our study revealed that invasion of opportunistic pathogens such as Clostridia and Synergistia might play a significant role in PoAP, thus guiding the further study of complex microbial-host interactions and the development of more effective diagnostic and therapeutic methods.

## Introduction

Apical periodontitis (AP) involves inflammatory destruction of periradicular tissues caused by a reaction of the host immune system to the presence of invaded microorganisms (in planktonic state or biofilms) or microbial products (Siqueira and Rôças, 2013; Shin et al., 2018). Root canal treatment is the most common method of treating AP. Post-treatment apical periodontitis (PoAP) occurs when root canal treatment has not adequately eliminated the infection, and its treatment represents a critical challenge in the clinic. According to numerous cross-sectional studies from various countries, the prevalence of PoAP in endodontically treated teeth is high, ranging from 24.1% to 53.5%, placing a heavy burden on medical resources (Liang et al., 2013; Di Filippo et al., 2014; Zhang et al., 2015). Furthermore, there is growing evidence that demonstrates associations between chronic apical periodontitis lesions and systemic diseases such as diabetes mellitus or cardiovascular diseases (Segura-Egea et al., 2015). Residual microbes that exist primarily in biofilms in the apical portion are thought to be the major cause of PoAP in properly treated cases (Nair, 2006). Consequently, it is necessary to uncover the pathogenic microorganisms and their functions associated with AP to clarify the disease process and provide a theoretical basis for more precise diagnosis and effective treatment.

Traditionally, bacteria have been studied using culture-based techniques, which rely on isolation, growth, and laboratory identification by morphological and biochemical tests. These studies revealed that in both acute and chronic AP, the microbiota is polymicrobial and predominantly anaerobic, harboring up to 12 species (Dahlén, 2017). Gram-positive and facultative anaerobic bacteria, such as *Streptococcus*, *Enterococcus*, *Lactobacillus*, *Propionibacterium*, and *Actinomyces* species, are considered the main persistent microorganisms in endodontically treated teeth (Molander et al., 1998; Chávez de Paz et al., 2003). However, researchers have shown that more than 50% of oral bacteria cannot be cultivated by routine laboratory techniques (Dewhirst et al., 2010). Thus, applying only cultural methods may lead to underestimating as-yet-uncultivated species (Zargar et al., 2020). Recently, the development of metagenomic sequencing has enabled us to gain increasing insight into the bacterial diversity of infected root canal systems. Some important periodontal pathogens, such as *Tannerella forsythia*, that have never been cultivated from infected root canals were detected in AP (Siqueira and Rôças, 2017). Furthermore, next-generation sequencing can aid in data analysis for thousands of different amplicons, rather than just individual or multiple samples, yielding enormous datasets, thereby offering more profound insight into AP (Park et al., 2020).

Microbial communities can vary their characteristics to adapt to environmental conditions for survival and maintenance (Clemente et al., 2012). Likewise, microbes could occupy different ecological niches with AP development, leading to dysbiosis, and exhibit qualitative and quantitative differences. For primary apical periodontitis (PAP), microorganisms typically colonize the pulp through caries lesions. While for PoAP, microorganisms are mainly located in the apical portion, resistant to the antimicrobial procedures and capable of enduring nutrient deprivation (Siqueira and Rôças, 2009). In a recent systemic review, Manoil et al. (2020) pointed out that all types of AP were correlated with a highly diverse microbiota. Yet the specific community distribution of each infection type remained controversial. Furthermore, due to the difficulty of microbial sampling in the apical portion niche, little is known about the pathogenesis of AP after root canal therapy. In recent years, the application of endodontic microsurgery in the treatment of PoAP has made it possible to obtain samples from the lesion area precisely. This paper explores the distinctive microbiota distribution from healthy oral to PoAP.

In this study, we use metagenomic analyses to characterize the differentiated composition of AP microbiotas at different stages, from healthy oral to PAP to PoAP. We also evaluated the differences in microbial metabolism in AP using KEGG annotation, providing a preliminary insight for further research on the complex microbial-host interaction and pathogenesis of AP.

## Material and methods

### Patient inclusion

The present study protocol was approved by the ethics board of Peking University Hospital of Stomatology, Beijing, China (no. PKUSSIRB-2013057). Patients with endodontically treated teeth that received endodontic microsurgery were included according to the following criteria from October 2019 to December 2020 at the Department of Cariology and Endodontics of the Peking University School of Stomatology.

The inclusion criteria were as follows:

1) The treated teeth had adequate coronal restoration.
2) True periapical lesions without involving the periodontal tissues (probing depth [PD] ≤ 3 mm).
3) Written informed consent.

The exclusion criteria were as follows:

1) A history of antibiotic use in the past 1 month.
2) Vertical root fracture were identified during surgery.
3) Resurgery teeth.

### Sample collection

Samples were obtained directly from apical lesions during endodontic microsurgery. Briefly, patients were anesthetized using 4% articaine with 1:100,000 epinephrine (Primacaine; Acteon Pharma, Bordeaux, France). Sulcular or mucogingival incisions were chosen depending on the tooth type and esthetic requirements of the case. Osteotomy was established with sterile fissure burs (Lindemann H161 Burs; Brasseler USA, Savannah, GA) under the application of sterilized water for cooling. Then, the infected granulation tissue was removed by a new sterile curette, part of which was biopsied and the remainder was collected in sterile PBS for subsequent analysis (SOFT samples). The root tip that was perpendicular to the long axis of the tooth was sectioned and collected similarly (HARD samples). All clinical procedures were performed by endodontists with at least five years of relevant experience. Before and during sampling, the tissue flap was pulled by an assistant to prevent contamination.

### DNA extraction and 16S rRNA gene amplicon sequencing

According to the manufacturer’s instructions, genomic DNA was extracted from oral samples using the DNeasy^®^ Blood & Tissue Kit (Cat No. 69504, Qiagen). The extracted DNA was quantified using NanoDrop 2000 spectrophotometer (ThermoFisher Scientific, Wilmington, Delaware, USA) and diluted to 20 ng/µL. The V3-V4 regions of 16S rRNA genes were amplified with the 341F/805R primers (CCTACGGGAGGCAGCAG/GACTACHVGGGTATCTAATCC). For amplicon sequencing, libraries were prepared with MiSeq library preparation Reagent Kit v3 (Illumina, USA) and then sequenced on a NovaSeq 6000 Sequencer platform (Illumina, USA) by Genesky Biotechnology Inc. (Shanghai, China).

### Data preparation and analysis

The raw sequencing reads of 16S rRNA genes of 10 unstimulated saliva samples from healthy oral (HO) conditions (SRA: DRP007410, submitted by Metabologenomics, Inc., Japan) and 10 infected dental pulp samples from primary AP (PAP) conditions (SRA: SRP121389, submitted by Institute Pasteur of Shanghai CAS, China) were downloaded from NCBI for further analysis. A total of 40 samples, including the 16S rRNA gene sequences from this study and the downloaded SRA datasets, were trimmed, and chimeric reads were filtered and assigned to operational taxonomic units (OTUs) at 97% identity using the UPARSE algorithm with USEARCH (Edgar, 2013). The taxonomy of each OTU was assigned using QIIME2 V2021.4 (Bolyen et al., 2019) with the SILVA132 database (Quast et al., 2018). The generated OTU table was normalized to 20,000 reads per sample used for the downstream analysis and visualization. Alpha and beta diversity indexes were calculated using the Vegan package in R (Oksanen et al., 2020). All color-scaled heatmaps were generated with the PHEATMAP R package (Kolde, 2019). Linear discriminant analysis (LDA) effect size (LEfSe) analysis was performed to identify taxa showing the most significant differences in microbial abundance between groups (Segata et al., 2011). Only taxa with LDA scores > 4.0 and p < 0.05 are shown. Pathway prediction for the oral microbiota of all samples was carried out with PICRUSt2. The normalized pathway abundance was visualized with a heatmap, and the discrepancy in significant pathways between groups was analyzed using Statistical Analysis of Metagenomic Profiles (STAMP) (Parks et al., 2014).

### Statistical analysis

Based on the ANOVA *F*-value of Shannon diversity, the power and sample size calculation were performed using the *pwr.anova.test()* function in the R package pwr (Xia et al., 2018). The Kruskal-Wallis one-way analysis of variance by ranks and Bonferroni t-test were performed to analyze the alpha-diversity differences between different sample types. Permutational multivariate analysis of variance (PERMANOVA) statistical analyses and pairwise tests were conducted based on the unweighted UniFrac matrix, and values were obtained using type III sums of squares with 999 permutations of residuals under a reduced model. The nonparametric factorial Kruskal-Wallis sum-rank test was used to determine the differential abundance in LEfSe analyses. In addition, Spearman’s correlation analysis was performed to calculate the correlation coefficient between metabolites and bacteria. Welch’s t-test was executed in STAMP to analyze the predicted functions of bacteria. For all statistical analyses, p < 0.05 was considered statistically significant.

## Results

The power analysis result showed that a sample size of more than 2 per group could provide more than 99% statistical power. Hense, a total of 10 patients (10 teeth) with a mean age of 35.5 years (range, 17-60 years) were enrolled in this cohort according to the inclusion/exclusion criteria. Clinical information was collected from the medical records and shown in **Table 1**

**Table 1:** Clinical information of Included patients (n = 10).

No.	Sex	Age	Tooth position	Symptom	Preoperative lesion size (CBCT, mm)	Quality of RCT (re-RCT) prior to surgery	Pathology diagnosis
1	Male	31	22	Swelling, Sinus	10.6×13.3×16.2	Satisfactory (RCT)	Granuloma
2	Female	37	12	Asymptomatic	10.1×10.2 (PA)	Satisfactory (RCT)	Granuloma
3	Male	26	41	Sinus	6.3×6.2×6.3	Satisfactory (RCT)	-
4	Female	29	36	Pain, Swelling	6.0×7.2×7.8	Satisfactory (re-RCT)	Granuloma
5	Male	34	12	Pain, Swelling	9.0×10.2×7.3	Satisfactory (RCT)	Cyst
6	Male	33	46	Pain, Swelling, Sinus	6.5×6.2×5.4	Unsatisfactory (RCT)	Granuloma
7	Female	17	21	Pain, Swelling	8.0×6.9×9.4	Unsatisfactory (RCT)	Granuloma
8	Female	60	27	Pain	7.9×8.1×5.6	Satisfactory (re-RCT)	Granuloma
9	Male	35	16	Pain	6.0×4.4×7.5	Satisfactory (RCT)	Granuloma
10	Female	58	21	Asymptomatic	3.0×3.7×5.4	Satisfactory (RCT)	Granuloma

- Preoperative lesion size: the largest dimension of bucco-lingual (BL), mesio-distal (MD), and vertical (V) diameter.

- Quality of RCT (re-RCT) prior to surgery:

Satisfactory root filling: 0-2 mm within the radiographic apex (flush) without viods.

Unsatisfactory root filling: either short (>2mm short of radiographic apex) or long (extrude beyond the radiographic apex) with or without voids or flush root fillings with voids.

### Overview of sequencing analysis

A total of 3,794,105 raw read pairs were generated by Illumina sequencing of the 16S rRNA V3-4 amplicon libraries. After quality control, including the removal of singleton sequences and chimeras, 3,316,784 high-quality sequences remained, with an average length of 400 bases. The rarefaction curves indicated that the species representation in each sample had approached the plateau phase, and it was unlikely that more microorganisms would be detected with additional sequencing efforts. These high-quality sequences were clustered into 2522 OTUs by the UPARSE pipeline using a threshold of 97% identity.

### Microbial community composition and structure succession analysis

**Figure 1:**
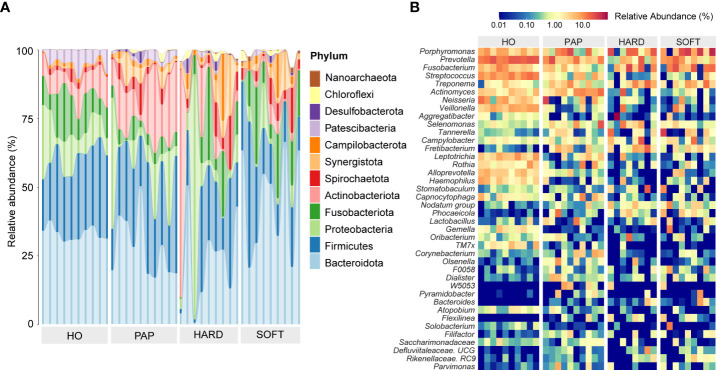
The phylogenetic profiling of microbiotas in healthy oral (HO), primary apical periodontitis (PAP), and persistent apical periodontitis (AP). Samples of persistent AP were classified into root apex (HARD) and periapical granulation tissues (SOFT). **(A)** Relative abundance of major microbial species at the phylum level. **(B)** Heatmap of relative abundance at the genus level.

A total of 29 phyla were detected across all samples, of which 12 phyla accounted for more than 99% of the entire microbial community, including Bacteroidota, Firmicutes, Proteobacteria, Fusobacteriota, Actinobacteria, Spirochaetota, Synergistota, Campilobacterota, Patescibacteria, Desulfobacterota, and Chloroflexi, as well as Nanoarchaeota belonging to Archaea. The relative abundance of microbial communities in different samples at the phylum level is shown in **Figure 1**. Among these phyla, Bacteroidota was the most abundant phylum, followed by Firmicutes, which together constituted more than 50% of the 32 samples. The relative abundance of Proteobacteria varied significantly in all samples, ranging from 0.1% to a maximum of over 89%. Other phyla, such as Verrucomicrobiota, Gemmatimonadota, and Halobacterota, constituted only a very small proportion (<1%) of the total microbial community.

**Figure 2:**
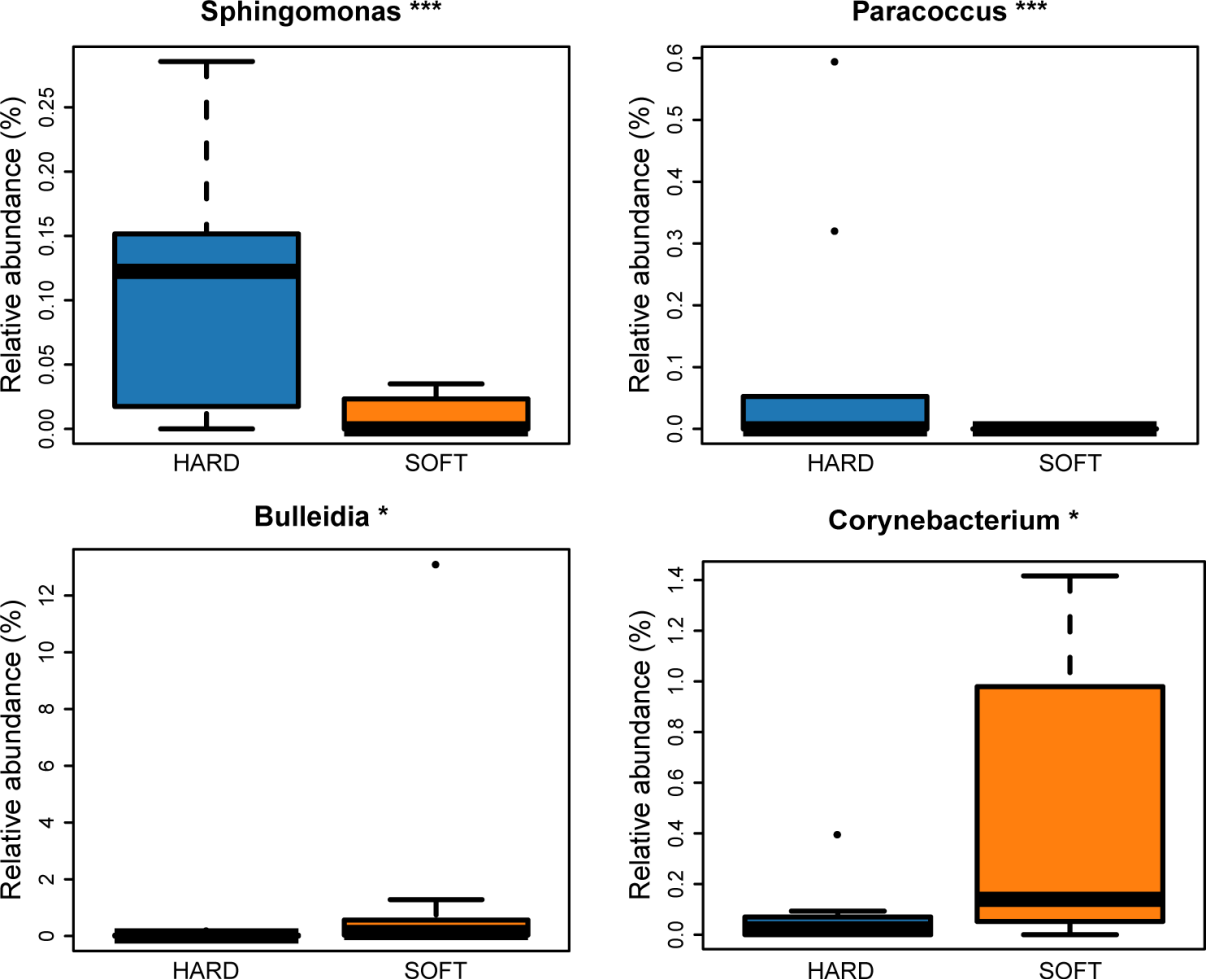
Microorganisms with significant difference between HARD and SOFT samples at the genus level. (*p < 0.05; ***p < 0.001).

According to the abundance heatmap at a detailed genus level (**Figure 1**), we can find that as the disease progressed, the relative abundance of some microbial taxa significantly decreased, such as *Prevotella, Streptococcus, Neisseria, Veillonella, Rothia, TM7x* and *Capnocytophaga* (**Supplementary Figure 1**), while *Porphyromonas, Fusobacterium, Treponema, Tannerella* gradually increase (**Supplementary Figure 2D**). Additionally, a relatively high abundance of *Actinomyces* was found in PAP samples (**Supplementary Figure 2**). When comparing the HARD and SOFT samples both from PoAP, it could be found that the relative abundance of *Sphingomonas* and *Paracoccus* significantly decreased in SOFT (**Figure 2**), whereas the abundance of *Bulleidia, Corynebacterium* increased (**Figure 2**).

**Figure 3:**
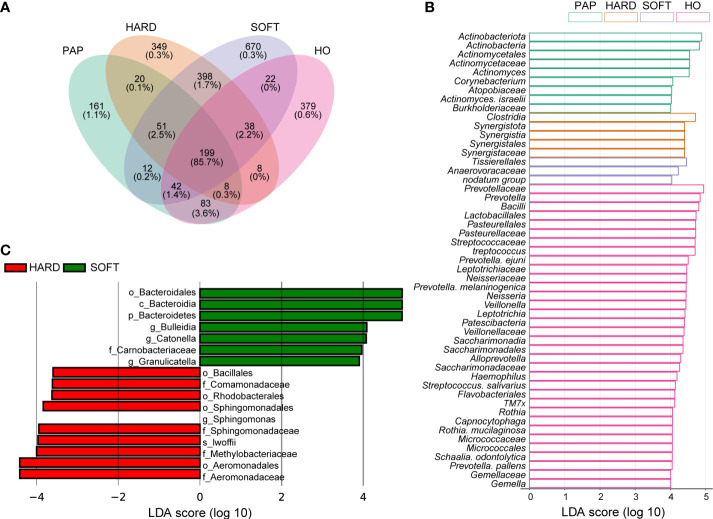
Distribution of the microbiota composition and biomarkers. **(A)** Venn diagram of the taxonomic distribution, the percentage under OTU numbers represent reads percentages of all sequencing data. **(B-C)** The result of linear discriminant analysis integrated with effect size (LEfSe). Microbial marker taxa with significant abundance differences among HO, PAP, HARD and SOFT **(B)** and between HARD and SOFT **(C)** (LDA > 3.5, p < 0.05).

Furthermore, Venn diagram analysis showed that most OTUs (86.5% of all sequence reads) were shared across all samples, and each type of sample also had its own specific taxa (**Figure 3**). Using LEfSe analysis, 51 microbial marker taxa that could distinguish HO samples from diseased samples were identified (LDA score > 4, **Figure 3**). Among them, *Actinobacteria* and *Actinomycetes* were especially prevalent in PAP samples, *Synergistales* were more frequently detected in HARD samples, while *Anaerovoracaceae*, *Tissierellales and nodatum group* were identified as microbial markers in SOFT samples. We also conducted LEfSe analysis to screen the potential biomarkers between HARD and SOFT. The results showed that the potential biomarkers of the SOFT were *Bulleidia, Catonella, Granulicatella*, etc. On the other hand, the potential biomarkers of the HARD were *Sphingomonas, lwoffii, Aeromonadaceae*, etc. (**Figure 3**).

### Microbial richness and diversity analysis

**Figure 4:**
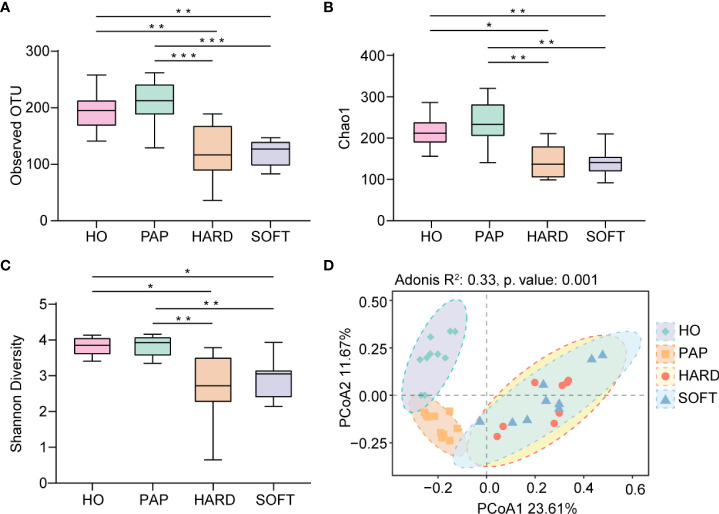
Comparison of diversity indices between HO and different stages of AP based on 16S rRNA sequencing data. **(A)** Observed OTUs, **(B)** Chao1 index, and **(C)** Shannon diversity index indicate the decrease of alpha diversity in persistent AP compared with HO and PAP. (*p < 0.05, **p <0.01, ***p <0.001) **(D)** Principal coordinates analysis (PCoA) of beta diversity based on unweighted UniFrac distances representing the differences among HO, PAP, and PoAP (HARD and SOFT). (Adonis R^2^ and p. value were generated by PERMANOVA).

Microbiota sequences were subsampled at 20,000 reads per sample prior to diversity assessments, resulting in a total of 38 samples meeting this threshold for further analysis. Microbial alpha diversity was assessed using three metrics: the observed OTUs (observed microbial richness), Chao1 index (estimated microbial richness), and Shannon diversity index (estimated evenness and richness). Compared to the HO and PAP samples, all diversity measures were decreased dramatically in the PoAP for both HARD and SOFT samples (**Figure 4**). Additionally, principal coordinates analysis (PCoA) of beta diversity was performed based on unweighted UniFrac distances. In the PCoA plot, a significant distinct microbiota pattern was observed within HO, PAP, and PoAP samples(**Figure 4**). On the other hand, the micerobiota of HARD and SOFT samples from PoAP shared similar community structures in pairwise comparison (**Table 2**).

**Table 2:** PERMANOVA pairwise comparison based on unweighted unifrac distance.

Comparison groups	R^2^	p.value	p.adjusted
HO vs PAP	0.41622061	0.001	0.0012
HO vs HARD	0.59470069	0.001	0.0012
HO vs SOFT	0.50084189	0.001	0.0012
PAP vs HARD	0.65767211	0.001	0.0012
PAP vs SOFT	0.54999313	0.001	0.0012
HARD vs SOFT	0.04821767	0.541	0.5410

### Metabolic pathway prediction

**Figure 5:**
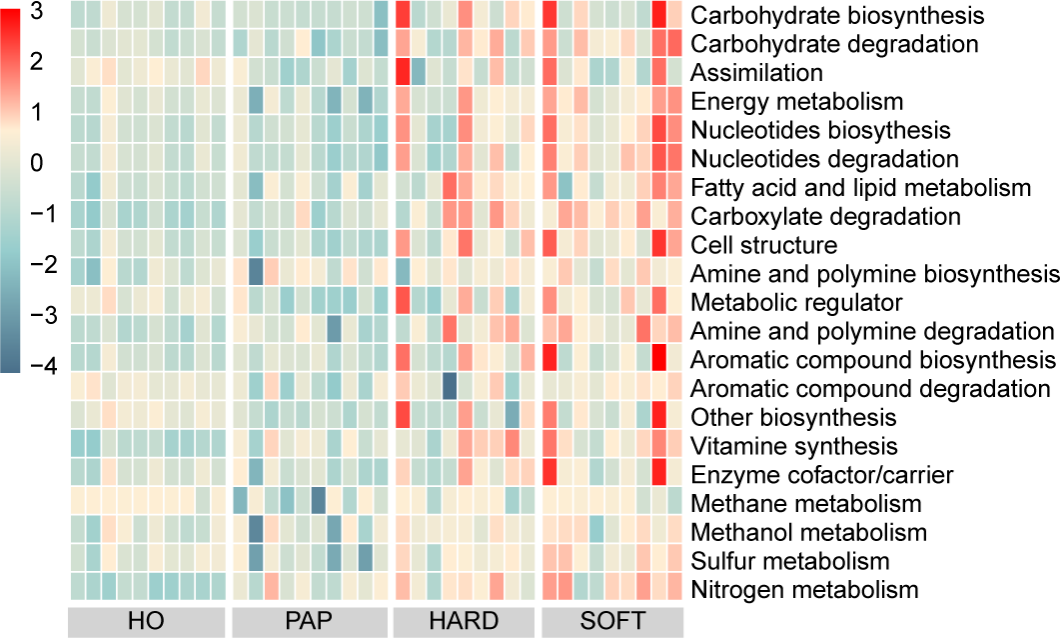
Heatmap showing differentially abundant metabolic pathways among HO, PAP, and persistent AP (HARD and SOFT). The abundance is predicted and normalized using PICRUSt2 with the default Metacyc database.

**Figure 6:**
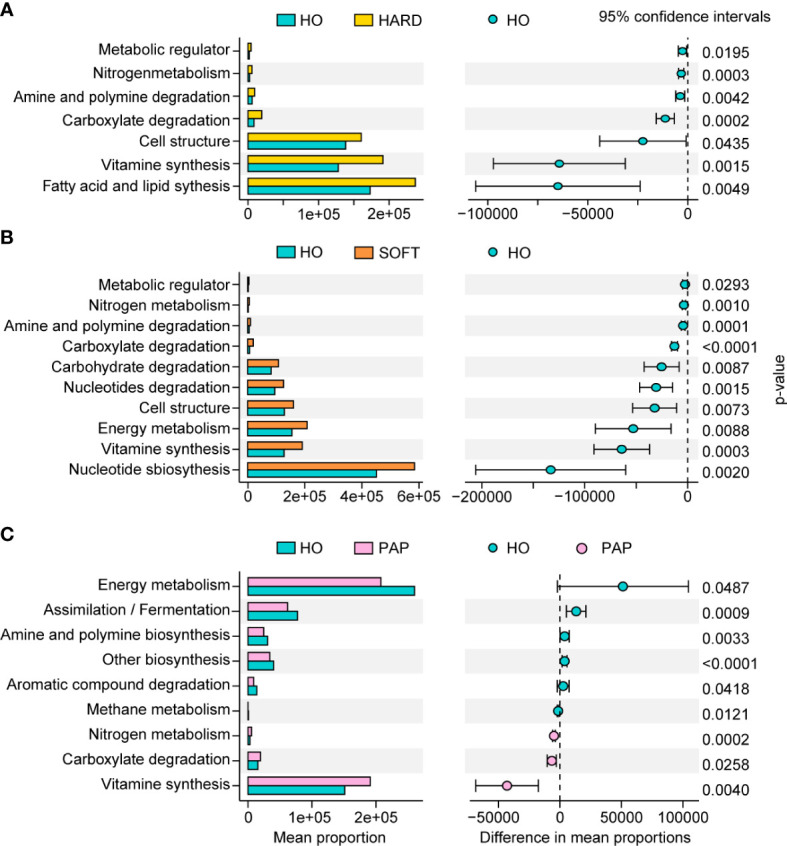
Metabolic pathway differences in microbiota in HARD **(A)**, SOFT **(B)** and PAP **(C)** samples compared with HO. The p values at the 95% confidence intervals indicated the potential activity differences of the metabolic pathways.

PICRUSt2 was used to elucidate the possible microbial metabolism among the PAP, PoAP (HARD and SOFT), and HO microbiotas. The colored scales show the normalized pathway abundance in each sample (**Figure 5**). The heatmap shows that microbiota in PoAP samples could have a more active metabolic capacity, including carbohydrate metabolism, energy metabolism, and enzyme cofactor biosynthesis. In addition, the metabolic processes of cell structure, vitamin synthesis, and fatty acid and lipid synthesis were more active in HARD samples of PoAP than in HO samples (**Figure 6**). In contrast, energy metabolism and nucleotide synthesis were more active in SOFT samples of PoAP than in HO samples (**Figure 6**). Besides, our analysis indicates that energy metabolism and assimilation/fermentation metabolic processes were depleted in PAP samples, while nitrogen metabolism and vitamin synthesis were enhanced (**Figure 4**).

## Discussion

The oral cavity is one of the most diverse microbial habitats in the human body, harboring approximately 1,000 microbial species (Wade, 2013). The use of metagenomic sequencing technologies could enable the characterization of not only the *in situ* composition and function of the microbiota but also variations across anatomic sites, time, and individuals (Huttenhower et al., 2012). AP is considered a biofilm-related oral disease (Siqueira et al., 2010). Microorganisms existing under PoAP conditions often form robust intra/extraradicular biofilms that are difficult to acquire by traditional paper point sampling (Siqueira and Rôças, 2009). In the present study, samples representing PoAP were obtained directly from the infected regions during endodontic microsurgery. These samples were classified as root apex (HARD) and periapical granulation tissues (SOFT) for the first time, facilitating a more precise characterization of the pathogenic microbes associated with PoAP. To understand the complex microbial signature at different developmental stages of AP, we coanalyze 16S rRNA sequencing data from clinical samples of PoAP together with datasets of HO and PAP available from the public database, providing a valuable reference for further research on the interaction mechanism of the microbial community in PoAP conditions.

As a relatively independent ecosystem, the oral cavity is considered to maintain a stable microecological structure. Zaura et al. (2009) reported that the predominant taxa in unrelated healthy individuals belong to Firmicutes, Proteobacteria, Actinobacteria, Bacteroidetes, and Fusobacteria, supporting the concept of the core oral microbiota. In the present study, these taxa were also shared between AP and HO samples (**Figure 1**), implying that the core microbiota is probably the best suited for the oral microenvironment and contributes significantly to the maintenance of oral function in both healthy and diseased states (Radaic and Kapila, 2021). Nevertheless, each periapical status still had a unique microbiota. From the perspective of microbial diversity, there was an obvious decrease in alpha-diversity indexes in PoAP (HARD and SOFT). This finding probably indicates that endodontic therapy could have disrupted the microbial balance and reduced both richness and diversity within samples. In contrast, the microbial diversity and richness of teeth with PAP are almost the same as those in HO samples (**Figure 4**). Meanwhile, the PERMANOVA test performed in this study allowed robust, unbiased analysis of multivariate data based on complex experimental designs and models (Anderson et al., 2008). PERMANOVA and pairwise comparison analyses return p-values for significance and R^2^ values, indicative of the amount of variation attributed to a specific treatment within a model. The results showed that the beta diversity of the samples was clearly divided into 3 clusters, which indicated the progress of the disease is closely associated with the microbial composition of AP (**Figure 4**; **Table 2**).

To unveil the microbial consortium specifically involved in PoAP, comparative phylogenetic profiling was performed using metagenomic sequencing datasets. The results showed that the relative abundance of some anaerobic marker bacteria species (LDA>4.0) exhibited upregulation (**Figure 1B** and **2D**), such as that of *Porphyromonas, Fusobacterium, Treponema, Prevotella, Peptostreptococcales*, and *Capnocytophaga.* Within these taxa, many species belong to the “red, orange, or green microbial complexes” of periodontal pathogens classified by Dr. Socransky (Socransky et al., 1998). Primarily, we observed the abundance increase of *Porphyromonas, Tannerella*, and *Treponema*, categorized within the “red complex” that might pose high pathogenicity linked to periodontal diseases. However, the relationship between the “red complex” and endodontic infection is still unclear. Gomes et al. (2007) found that the “red complex” in endodontic infection was associated with tenderness to percussion and pain on palpation, while Rôças et al. did not find any correlation between clinical signs and the presence of *T. forsythia* (Rôças et al., 2001). The other upregulated microbial markers in PoAP, such as *Clostridia* and *Synergistia*, have also been recently found in other studies as pathogens of periodontal disease, gastrointestinal infections, or soft tissue infections (Hiranmayi et al., 2017). However, their specific pathogenic mechanisms and their relationship with PoAP remain to be further investigated. Our analysis indicated that the “red complex” species, as well as other emerging periodontal pathogens, could be associated with the pathogenesis of PoAP.

Additionally, it can be found that although both HARD and SOFT samples were derived from PoAP patients, microorganisms shows an obvious preference between them (**Figure 2**). The comparative analysis of the two reveals that the genus *Bulleidia* and *Corynebacterium* were the indicators of SOFT samples. According to Morgan and Goldstein (2021), *Bulleidia* is an important dental pathogen and can cause distant site infections (e.g. prosthetic joints). However, this pathogen is challenging to isolate due to its fastidious culture requirements and identification demands. Thus, there is no systematic, supportive susceptibility information about *Bulleidia*. Clinicians and microbiologists need to be aware of its extended pathogenic potential and explore more data about this emerging pathogen. In the HARD samples, the identified indicators included *lwoffii, Sphingomonas*, etc. Some of these bacteria are capable of enduring poor nutrients and environmental stress, which has been identified as the cause of nosocomial infections (Cao et al., 2018; Asaf et al., 2020).

In contrast to PoAP and HO, most of the upregulated microbial markers in primary AP belonged to *Actinomyces*, which is considered part of the oral flora and has the ability to adhere to the oral tissue and thereby resist cleansing mechanisms. Some studies have demonstrated that *Actinomyces* spp. play an essential role in the formation of dental biofilms and could contribute to the development of diseases such as caries and periodontitis (de Oliveira et al., 2020). Dioguardi et al. (2020) have previously reviewed microbial associations with Actinobacteria in primary endodontic lesions, which indicated the selective conditions of anaerobiosis and the loss of the integrity of the oral mucous membrane could give rise to infection by these microorganisms. In addition, other microbial markers, such as *Lactobacillus, TM7*, and *Rothia*, were significantly depleted in the AP group compared with the HO group. Many *Lactobacillus* species have been considered probiotics, which can maintain or improve microbial homeostasis in the host environment and inhibit pathogen invasion and colonization (Zhang et al., 2018). Therefore, the use of probiotics to restore microbial homeostasis may provide new ideas for precision therapy.

To elucidate the mechanism of pathogenicity of microorganisms in AP, we performed preliminary KEGG metabolic predictions by linking the 16S rRNA data with the functional annotation of sequenced prokaryotic genomes. Compared to the HO and PAP samples, the microbiota of the PoAP (HARD and SOFT) samples showed a more active metabolic capacity. In addition, several processes of bacterial biosynthesis, such as nucleotide metabolism, carbohydrate biosynthesis, fatty acid and lipid synthesis, and enzyme cofactor/carrier processes, were enhanced. These findings reflect that the microorganisms associated with PoAP could be more pathogenic.

While in the present study, our analyses were based on high-throughput DNA sequencing, which raises the common question of whether the bacteria identified were still alive since NGS protocols also detect free DNA originating from dead cells (Nair, 2007). Although some researchers have attempted to address this problem by removing free DNA before nucleic acid extraction, the reliability of these methods has not been extensively verified (Nocker et al., 2006; Villarreal et al., 2013). There is also evidence that the longevity of free DNA in the infected root canal could be short and therefore unlikely to represent a major proportion of the DNA isolated (Brundin et al., 2010). Besided, it is well known that apical periodontitis is a dynamic process with distinct pathogenetic stages, so even detection of DNA from dead cells can be of interest to our understanding of the ecological structure of the periapical microbiota (Siqueira, 2008). In addition, our functional analysis based on PICRUSt and KEGG Mapper is just a prediction; further researches are warranted to demonstrate our speculation and clarify the pathogenesis of apical periodontitis. Another potential bias in this study may originate from the contamination during obtaining or handling the specimen. For this instance, we performed adequate sterilization and continued to pull the tissue flap before and during sampling to prevent contamination; but a sterile control still needs to be taken.

## Conclusion

The present study characterized the microbial distribution and variation in PoAP compared with healthy oral and primary AP. In addition, we identified the PoAP-associated microbial consortium and marker taxa. We preliminarily predicted their metabolic differences, which might be associated with the etiology and pathogenesis of PoAP. These findings will provide essential guidance for clinical diagnostics, preventive intervention, and therapeutic management for PoAP. Future research will focus on comprehensive multi-omics studies of AP microbiotas at different stages and characterization of microbial-host interaction of the core pathogenic bacteria to study how they drive the destruction of the soft tissue and bone in PoAP.

## Data availability statement

The raw sequencing data of this study are available in the NCBI Sequence Read Archive with the accession number PRJNA808987.

## Ethics statement

The studies involving human participants were reviewed and approved by Institutional Review Board (IRB) of Peking University Hospital of Stomatology. Written informed consent to participate in this study was provided by the participants’ legal guardian/next of kin. Written informed consent was obtained from the individual(s), and minor(s)’ legal guardian/next of kin, for the publication of any potentially identifiable images or data included in this article.

## Author contributions

J-LZ conceptualized and designed the study, performed experiments, analyzed the data, and wrote the manuscript. JLY analyzed the data and helped with the polishing of the manuscript. LY helped with the insightful discussions. Y-HL and WBD conceptualized the study and critically revised the manuscript. All authors gave final approval and agree to be accountable for all aspects of the work.

## Funding

This work was supported by the National Key Research and Development Program of China under Grant (2021YFC2301000 and 2021YFA0717000) and the National Natural Science Foundation of China under Grant (21822408 and 81991501).

## Acknowledgments

We thank all the subjects who made this study possible.

## Conflict of interest

The authors declare that the research was conducted in the absence of any commercial or financial relationships that could be construed as a potential conflict of interest.

## Publisher’s note

All claims expressed in this article are solely those of the authors and do not necessarily represent those of their affiliated organizations, or those of the publisher, the editors and the reviewers. Any product that may be evaluated in this article, or claim that may be made by its manufacturer, is not guaranteed or endorsed by the publisher.
