# Supplementary material for: Distinctive microbiota distribution from healthy oral to post-treatment apical periodontitis
Source: Front Cell Infect Microbiol. 2022 Sep 8;12:980157. doi: 10.3389/fcimb.2022.980157 (PMC9492884; doi:10.3389/fcimb.2022.980157)
Supplement: Supplementary file 1 [file DataSheet_1.docx]

Supplementary Material

# Supplementary Figures

#
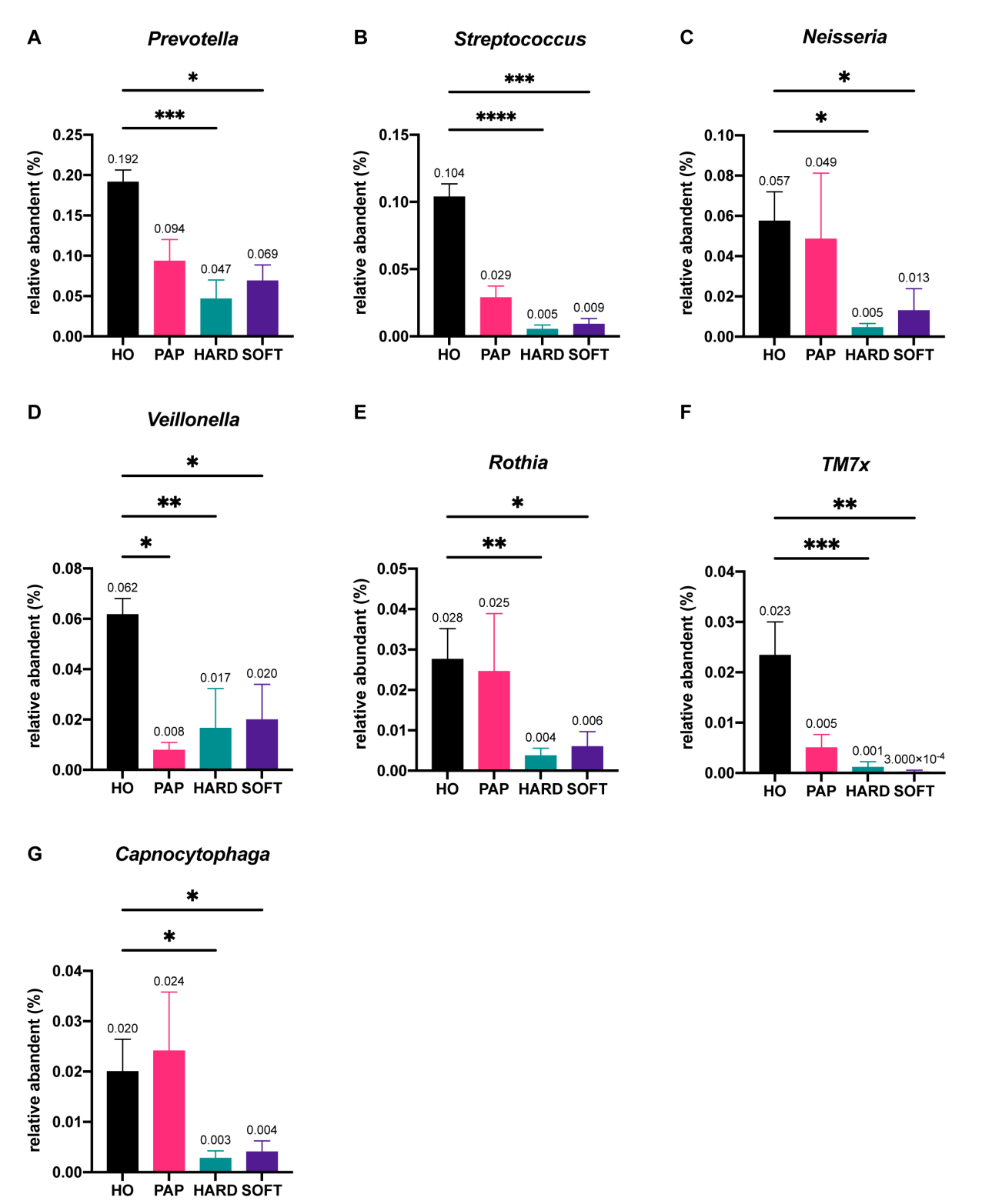


**Supplementary Figure 1.** The relative abundance of major microbial taxa showed a significant downward trend at the genus level.


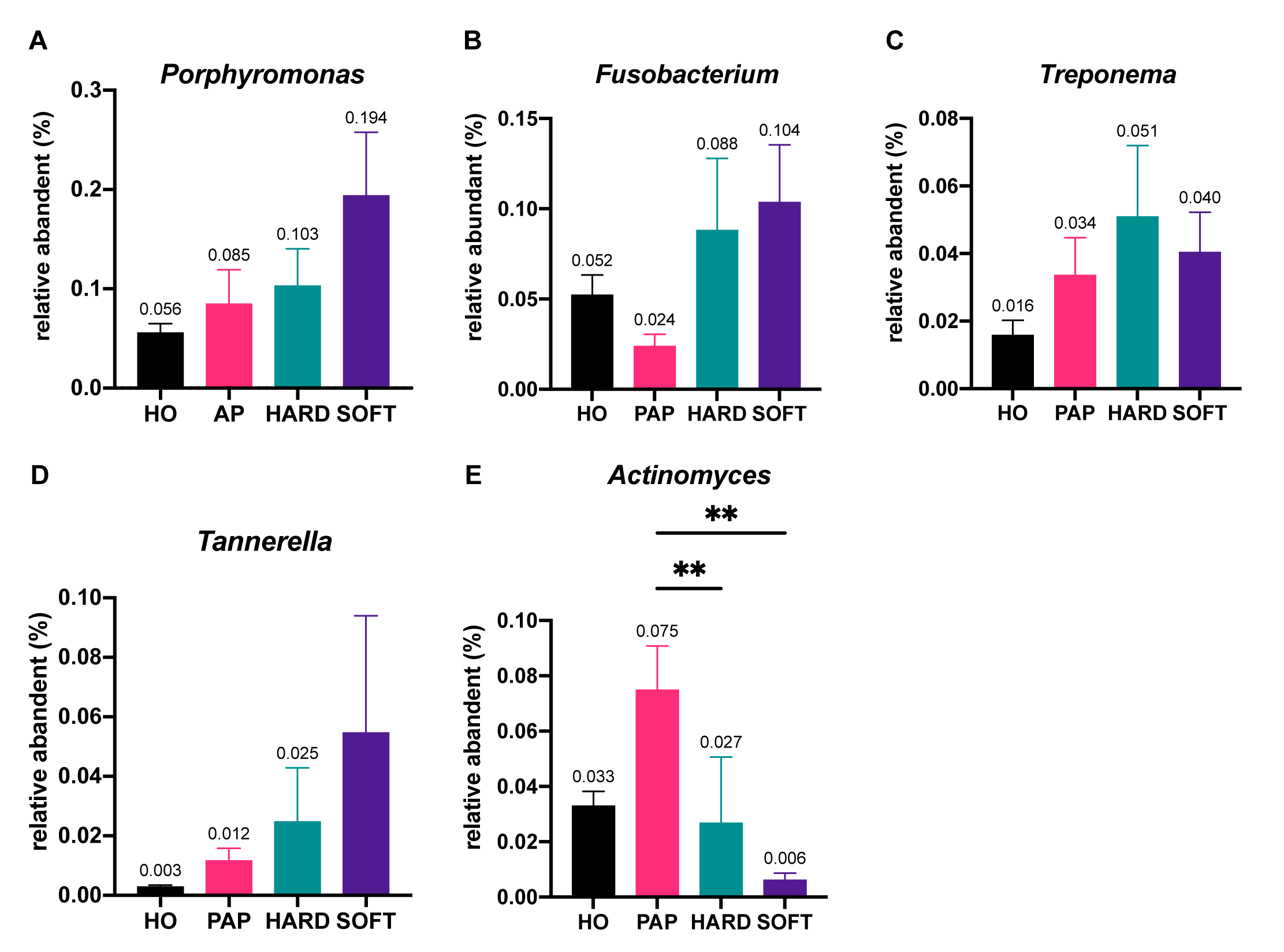


**Supplementary Figure 2.** (A-D) The relative abundance of major microbial taxa gradually increaseed at the genus level. (E) *Actinomyces* with relatively high abundance in PAP samples.
